# Palmitoylethanolamide/Baicalein Regulates the Androgen Receptor Signaling and NF-κB/Nrf2 Pathways in Benign Prostatic Hyperplasia

**DOI:** 10.3390/antiox10071014

**Published:** 2021-06-24

**Authors:** Ramona D’Amico, Tiziana Genovese, Marika Cordaro, Rosalba Siracusa, Enrico Gugliandolo, Alessio Filippo Peritore, Livia Interdonato, Rosalia Crupi, Salvatore Cuzzocrea, Rosanna Di Paola, Roberta Fusco, Daniela Impellizzeri

**Affiliations:** 1 Department of Chemical, Biological, Pharmaceutical, and Environmental Science, University of Messina, 98166 Messina, Italy; rdamico@unime.it (R.D.); tgenovese@unime.it (T.G.); rsiracusa@unime.it (R.S.); aperitore@unime.it (A.F.P.); livia.interdonato@yahoo.it (L.I.); rfusco@unime.it (R.F.); dimpellizzeri@unime.it (D.I.); 2 Department of Biomedical and Dental Sciences and Morphofunctional Imaging, University of Messina, 98166 Messina, Italy; cordarom@unime.it; 3 Department of Veterinary Science, University of Messina, 98166 Messina, Italy; egugliandolo@unime.it (E.G.); rcrupi@unime.it (R.C.)

**Keywords:** benign prostatic hyperplasia, androgen receptor, palmitoylethanolamide, baicalein, inflammation, oxidative stress

## Abstract

Benign prostatic hyperplasia (BPH) is the most common benign tumor in males. Androgen/androgen receptor (AR) signaling plays a key role in the development of BPH; its alterations cause an imbalance between prostate cell growth and apoptosis. Furthermore, chronic inflammation and oxidative stress, which are common conditions in BPH, contribute to disrupting the homeostasis between cell proliferation and cell death. With this background in mind, we investigated the effect of ultramicronized palmitoylethanolamide (um-PEA), baicalein (Baic) and co-ultramicronized um-PEA/Baic in a fixed ratio of 10:1 in an experimental model of BPH. BPH was induced in rats by daily administration of testosterone propionate (3 mg/kg) for 14 days. Baic (1 mg/kg), um-PEA (9 mg/kg) and um-PEA/Baic (10 mg/kg) were administered orally every day for 14 days. This protocol led to alterations in prostate morphology and increased levels of dihydrotestosterone (DHT) and of androgen receptor and 5α-reductase expression. Moreover, testosterone injections induced a significant increase in markers of inflammation, apoptosis and oxidative stress. Our results show that um-PEA/Baic is capable of decreasing prostate weight and DHT production in BPH-induced rats, as well as being able to modulate apoptotic and inflammatory pathways and oxidative stress. These effects were most likely related to the synergy between the anti-inflammatory properties of um-PEA and the antioxidant effects of Baic. These results support the view that um-PEA/Baic should be further studied as a potent candidate for the management of BPH.

## 1. Introduction

Benign prostatic hyperplasia (BPH) is a common chronic disease among men, and its incidence increases with age [1]. There are many potential etiological factors contributing to BPH pathogenesis such as an imbalance between prostate cell growth and apoptosis. Apoptosis is importantly involved in maintaining tissue homeostasis and controlling cell proliferation [2]. This programmed cell death entails a series of molecular events eventually resulting in the death and removal of infected or damaged cells while preventing the release of harmful substances into surrounding tissues [3]. Chronic prostatic inflammation was revealed to be closely associated with the occurence of BPH. Inflammatory stimuli appear to alter the homeostasis between cell proliferation and cell death, resulting in an increase in proliferative processes and a reduction in apoptotic cell death [4,5]. Moreover, prostatic inflammation generating free radicals exacerbates the damage. Macrophage and neutrophil infiltration produces a source of free radicals, which have been related to harmful oxidative effects on tissue and DNA [6]. Free radical formation can alter protein structure and function, induce gene changes and cause post-translational modifications including those involved in DNA repair and apoptosis. The molecular pathological mechanisms leading to BPH are still unclear; however, at present, testosterone and dihydrotestosterone (DHT) are known to be highly correlated with BPH. Testosterone, which is produced in the testes and spreads to the prostate, is converted into DTH by the action of 5α-reductase (5α-red 2) [7]. Possessing greater affinity for androgen receptor (AR) than testosterone, DHT is an acute mediator of BPH. The binding of DHT-AR complexes regulates the expression of target genes, leading to biological responses that include the metabolism, morphology, differentiation, proliferation and survival of prostate cells [8]. Indeed, it has also been well documented that the AR crosstalks with transforming growth factor-β (TGF-β), and that there is a correlation of higher TGF with rising serum prostate-specific antigen (PSA) levels in metastatic prostate cancer [9,10].

Currently, the most common medications used for BPH are 5α-red 2 inhibitors and α-blockers [1]. The 5α-red 2 inhibitors can reduce DHT levels, while the α-blockers relieve BPH symptoms by relaxing the smooth muscles in the prostate. However, 5α-red 2 inhibitors and α-blockers show several side effects, such as dizziness, erectile dysfunction and cardiovascular risks [11]. Therefore, developing novel strategies is imperative for the management of BPH.

In this regard, in recent years, palmitoylethanolamide (PEA) has aroused particular interest for its anti-inflammatory properties [12,13,14]. PEA, a fatty acid amide produced on demand after tissue damage, presents a lipid structure and a large size of heterogeneous particles; therefore, it may be expected to have limitations in terms of solubility and bioavailability [15,16]. Micronization and ultramicronization methods are applied to reduce the particle size (<10 μm) and represent a potential solution for bypassing this problem [17,18]. However, PEA does not have direct antioxidant action to prevent oxidative stress and counteract injury to proteins and DNA. Current studies have proven that baicalein (Baic), a flavonoid extracted from the root of Scutellaria baicalensis, shows antioxidant properties via free radical scavenging capacity and/or enhancing the antioxidant system [19,20]. Until now, multiple biological functions of Baic have been discovered. It has been shown that Baic has been widely used to treat various inflammatory diseases including cardiovascular diseases, chronic hepatitis and some selective cancers including breast cancer, hepatocellular carcinoma, leukemia and colon cancer [21,22,23].

Therefore, the objective of this research was to determine if the effect of the new co-ultramicronized compound, which combines the anti-inflammatory effect of ultramicronized PEA (um-PEA) with the antioxidant capacity of Baic in a fixed ratio of 10:1, can modulate AR signaling and reduce inflammation and oxidative stress associated with BPH.

## 2. Materials and Methods

### 2.1. Animals

Sprague Dawley rats (male, 200–250 g, Envigo, Italy) were accommodated in a controlled location and received food and water ad libitum. The University of Messina’s Review Board for Animal Care approved this study. Animal care conformed to Italian regulations on the use of animals for experimental and scientific purposes (D.Lgs 2014/26 and EU Directive 2010/63).

### 2.2. BPH Induction and Drugs

BPH was induced in animals by subcutaneous administration of testosterone propionate at the dose of 3 mg/kg, diluted in corn oil in a volume of 100 μL and administered for 14 days [24]. Animals were killed under anesthesia at the end of the experiment, and ventral lobes were preserved in 10% neutral buffered formalin and embedded in paraffin for histological studies. The remaining portions of each prostate were stored at −70 °C and used for further analyses.

Baic, um-PEA and the co-ultramicronized um-PEA/Baic were kindly provided from Epitech Group SpA (Saccolongo, Italy) and dissolved in carboxymethylcellulose (1% wt/vol in saline). Unless otherwise mentioned, all compounds were purchased from Sigma-Aldrich Company Ltd. (St. Louis, MO, USA).

### 2.3. Experimental Groups

Rats were randomly allocated into the following groups (*n* = 12 for each group):

BPH: rats were subjected to the above-described testosterone administration;

BPH + Baic: same as the BPH group, and Baic (1 mg/kg, o.s.) was administered for 14 days;

BPH + um-PEA: same as the BPH group, and um-PEA (9 mg/kg, o.s.) was administered for 14 days;

BPH + um-PEA/Baic (10:1): same as the BPH group, and um-PEA/Baic (10 mg/kg, o.s.) was administered for 14 days;

Sham operated groups: vehicle (carboxymethylcellulose (1% wt/vol in saline)) or Baic, um-PEA or um-PEA/Baic was orally administered for 14 days. Since no significant histological and macroscopic changes were found between the groups, we present data of the sham+vehicle group.

The doses and route of administration were chosen based on our previous studies [25,26,27].

### 2.4. Prostate Weight

Prostates were collected and weighed, and the ratio of growth inhibition was calculated as follows Equation (1):

Ratio of growth inhibition = 100 − [(TG (treated group) − Sham)/(BPH − Sham) × 100](1)

where TG is the values of the treated groups [24].

### 2.5. Histology

Ventral lobe sections of the prostate (7 μm) were stained with hematoxylin/eosin (H/E) for histopathological examination using a Leica DM6 microscope, associated with the Leica LAS X Navigator software (Leica Microsystems SpA, Milan, Italy) [28]. Every piece was observed at a magnification of 20X, and morphological changes were evaluated by two blinded investigators. The scoring system was chosen based on previous studies [24].

### 2.6. Staining of Mast Cells

Fourteen days after BPH induction, prostate tissue sections were stained with toluidine blue as described previously [29,30] to identify mast cells. Every section was observed at a magnification of 100X.

### 2.7. Western Blot Analysis of 5α-red 2, AR, PSA, TGF-β, Bax, Bcl2, IκBα, Nuclear Factor kappaB (NF-κB), Nrf-2, Heme Oxigenase-1 (HO-1) and Mn-SOD

Western blot analysis was performed on prostate samples (lateral lobes) as previously described [31,32]. The following primary antibodies were used: anti-5α-red 2 (1:500, Santa Cruz Biotechnology (SCB)), anti-AR (1:500, SCB), anti-PSA (1:500, SCB), anti-TGF-β (1:1000, Cell Signalling), anti-Bax (1:1000, SCB, #sc7480), anti-Bcl-2 (1:1000, SCB, #sc7382), anti-IκBα (1:1000, SCB, #sc1643), anti-NF-κB p65 (1:500, SCB, #sc8008), anti-Nrf2 (1:500; SCB, #sc365949), anti-HO-1 (1:1000; SCB), anti-MnSOD (1:1000, SCB, #sc137254), anti-β-actin (1:5000; SCB, #sc8432) and anti-lamin A/C antibody (1:5000; Sigma-Aldrich, St. Louis, MO, USA) [33]. Protein expression was quantified by densitometry with BIORAD ChemiDocTM XRS+ software and normalized to housekeeping genes β-actin and lamin A/C as previously reported [34,35].

### 2.8. Evaluation of Testosterone and DHT Levels in Serum and Prostate

The prostate tissue samples were homogenized (1/10 *w/v*) using a homogenizer in a tissue lysis/extraction reagent containing a protease inhibitor cocktail (Sigma-Aldrich; Merck Millipore, Darmstadt, Germany). The homogenates were centrifuged at 12,000× *g* for 25 min at 4 °C, and the protein concentration in the supernatant fractions was determined using the Bradford reagent (Bio-Rad Laboratories, Inc., Hercules, CA, USA). Testosterone and DHT levels were assayed in the serum and prostate tissue with a commercially available enzyme-linked immunosorbent assay (ELISA) kit (My BioSource, San Diego, CA, USA). The values were expressed as milligram protein and milliliters for the prostate and serum, respectively [36].

### 2.9. Evaluation of Cytokine Levels

Prostate levels of IL-1β, IL-6 and TNF-α were measured by ELISA kits (R&D Systems, Minneapolis, MN, USA). All protocols were performed by following the manufacturer’s instructions [37,38]. Absorbency was measured using a microplate reader at 450 nm. The concentration of the cytokines in the tissue was mentioned as protein in pg/mg.

### 2.10. Statistical Evaluation

All values are expressed as mean ± standard error of the mean (SEM) of N observations. The images shown are representative of the least 3 experiments conducted on diverse experimental days on tissue sections obtained from all animals in each group. For in vivo investigations, the number of animals utilized in in vivo investigations is denoted by the letter N. The results were analyzed by one-way ANOVA followed by a Bonferroni post hoc test for multiple comparisons. A *p-*value less than 0.05 was considered significant.

## 3. Results

### 3.1. Effect of um-PEA/Baic on Testosterone and DHT levels and 5α-red 2, AR and PSA Expression

**Figure 1:**
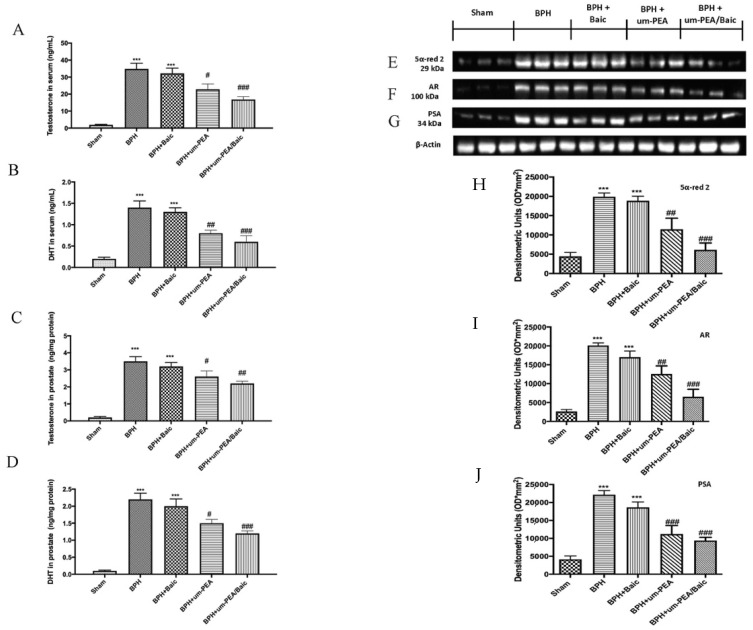
Effect of um-PEA/Baic on testosterone and DHT levels and 5α-red 2, AR and PSA expression. Testosterone levels in serum (**A**); DTH levels in serum (**B**); testosterone levels in prostate (**C**); DTH levels in prostate (**D**); Western blots and densitometric analysis of 5α-red 2 (**E**,**H**), AR (**F**,**I**) and PSA (**G**,**J**). A demonstrative blot of lysates with a densitometric analysis for all animals is shown. Values shown are means ± SEM of six animals in each group. *** *p* < 0.001 vs. sham; # *p* < 0.05 vs. BPH group; ## *p* < 0.01 vs. BPH group; ### *p* < 0.001 vs. BPH group.

It is known that testosterone is converted into DHT by the action of 5α-red 2. Therefore, we evaluated levels of testosterone and DHT both in serum and in prostate tissues. Serum levels of testosterone (Figure 1A) and DHT (Figure 1B) levels were markedly elevated in BPH rats and after treatment with Baic alone. Daily administration of um-PEA and even more um-PEA/Baic considerably reduced the levels of both androgens, comparable to the BPH group (Figure 1A,B). Similar results were observed also in prostate homogenates, as shown in panel 1C for testosterone and 1D for DHT levels.

Additionally, we evaluated 5α-red 2 expression in all groups by Western blot analysis. Basal expression of 5α-red 2 was detected in prostate tissue from sham animals; on the contrary, it was significantly increased in the BPH group. The oral treatment with um-PEA/Baic at 10 mg/kg, more than um-PEA alone at 9 mg/kg, substantially reduced 5α-red 2 expression. Baic at 1 mg/kg did not produce any significant reduction (Figure 1E,H).

Further, AR and PSA, which play an essential role in prostate development, were examined by Western blot. Consistently, AR (Figure 1F,I) and PSA (Figure 1G,J) expression were elevated after BPH induction compared to the sham group. Daily treatment with um-PEA was able to reduce both AR and PSA levels, but um-PEA/Baic showed a better effect compared to um-PEA alone. Conversely, Baic at 1 mg/kg did not show any significant changes compared to vehicle.

### 3.2. Effect of um-PEA/Baic on Cell Growth and Apoptotic Pathway

**Figure 2:**
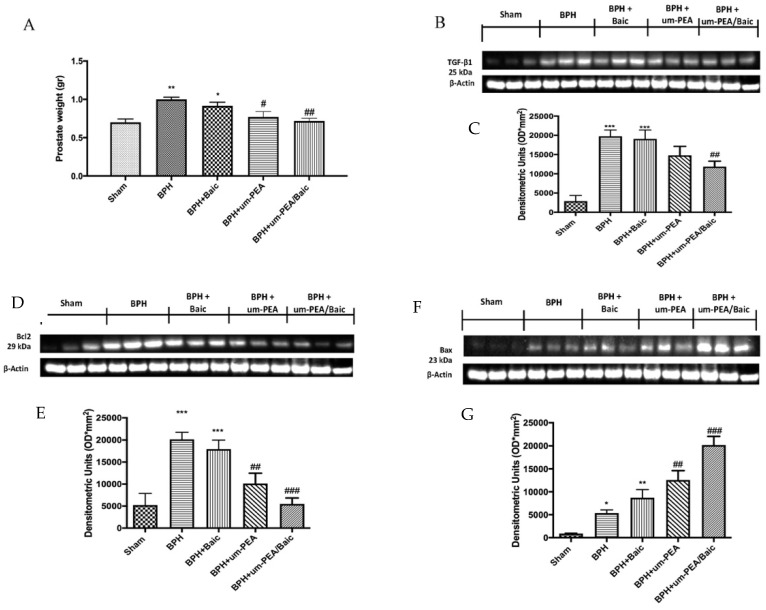
Effect of um-PEA/Baic on cell growth and apoptotic pathway. Prostate weight (**A**); Western blots and densitometric analysis of TGF-β (**B**,**C**), Bcl-2 (**D**,**E**) and BAX (**F**,**G**). A demonstrative blot of lysates with a densitometric analysis for all animals is shown. Values shown are means ± SEM of six animals in each group. * *p* < 0.05 vs. sham; ** *p* < 0.01 vs. sham; *** *p* < 0.001 vs. sham; # *p* < 0.05 vs. BPH group; ## *p* < 0.01 vs. BPH group; ### *p* < 0.001 vs. BPH group.

A disparity between prostate cell growth and apoptosis has been linked to BPH. Our results show an evident increase in prostate weight (Figure 2A) and TGF-β expression (Figure 2B,C) in BPH-treated rats which um-PEA/Baic (10 mg/kg) treatment significantly reduced.

To assess the anti-apoptotic effect of um-PEA/Baic, we observed the expression of the the anti-apoptotic Bcl-2 (Figure 2D,E) and pro-apoptotic Bax (Figure 2F,G) proteins by Western blot analysis. The finding shows a significant increase in Bcl-2 and Bax levels after BPH induction compared to control. Um-PEA/Baic at the dose of 10 mg/kg significantly reduced levels of Bcl-2 and increased the expression of Bax. Similar results were observed after um-PEA administration, but in a lower manner. On the contrary, Baic treatment did not show significant changes compared to vehicle.

### 3.3. Effect of um-PEA/Baic on Prostate Morphology

**Figure 3:**
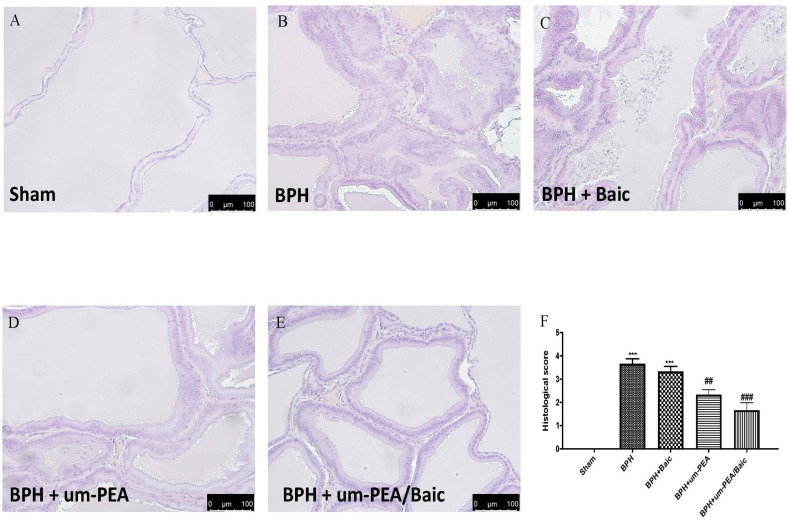
Effect of um-PEA/Baic on prostate morphology. Histological evaluation of the prostate: sham (**A**); BPH (**B**); BPH + Baic (**C**); BPH + um-PEA (**D**); BPH + um-PEA/Baic (**E**); histological score (**F**). Images are indicative of at least 3 independent experiments. Values shown are means ± SEM of six animals in each group. A 20X magnification is shown (100-µm scale bar). *** *p* < 0.001 vs. sham; ## *p* < 0.01 vs. BPH group; ### *p* < 0.001 vs. BPH group.

H/E staining showed a significant alteration of prostate tissue, an intense hyperplasia and a substantial inflammation after BPH (Figure 3B,F), while a normal architecture of the prostate with abundant acini was observed in sham animals (Figure 3A,F). Baic (1 mg/kg) treatment did not decrease tissue damage (Figure 3C,F). Oral administration of um-PEA/Baic (10 mg/kg) showed a significant reduction in tissue damage and hyperplasia compared to the vehicle group (Figure 3E,F). Additionally, um-PEA/Baic reduced histological alterations more effectively than the treatment with um-PEA (9 mg/kg) alone (Figure 3D,F).

### 3.4. Effect of um-PEA/Baic on Mast Cell Density

**Figure 4:**
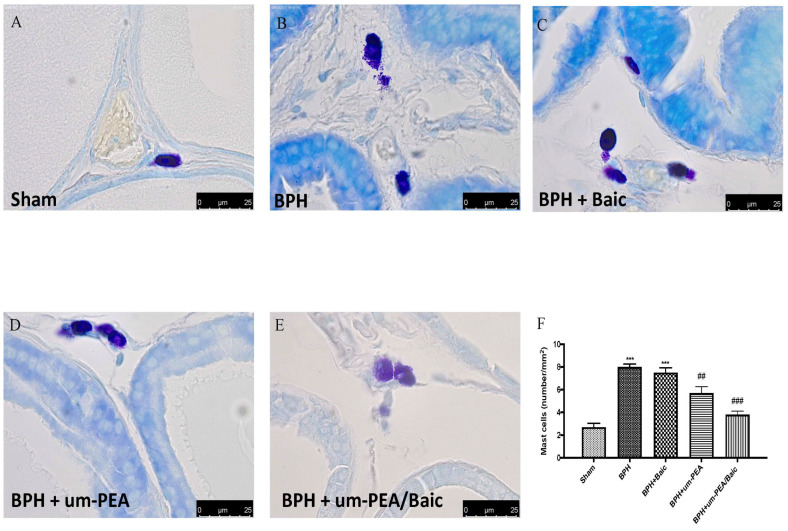
Effect of um-PEA/Baic on mast cell density. Evaluation of mast cell degranulation by toluidine blue: sham (**A**); BPH (**B**); BPH + Baic (**C**); BPH + um-PEA (**D**); BPH + um-PEA/Baic (**E**); mast cell count (**F**). Images are indicative of at least 3 independent experiments. Values shown are means ± SEM of six animals in each group. A 100X magnification is shown (25-µm scale bar). *** *p* < 0.001 vs. sham; ## *p* < 0.01 vs. BPH group; ### *p* < 0.001 vs. BPH group.

Toluidine blue staining was used to visualize the mast cell infiltration in the stromal cells of all the experimental groups. BPH resulted in an increased number of mast cells (Figure 4B,F) when compared to the control group (Figure 4A,F). The um-PEA group showed lower mast cell infiltration (Figure 4D,F) when compared to the BPH group, although the infiltration was higher when compared to that in the um-PEA/Baic group (Figure 4E,F). Baic treatment did not reduce the number of activated mast cells (Figure 4C,F).

### 3.5. Effect of um-PEA/Baic on Inflammation Pathway

**Figure 5:**
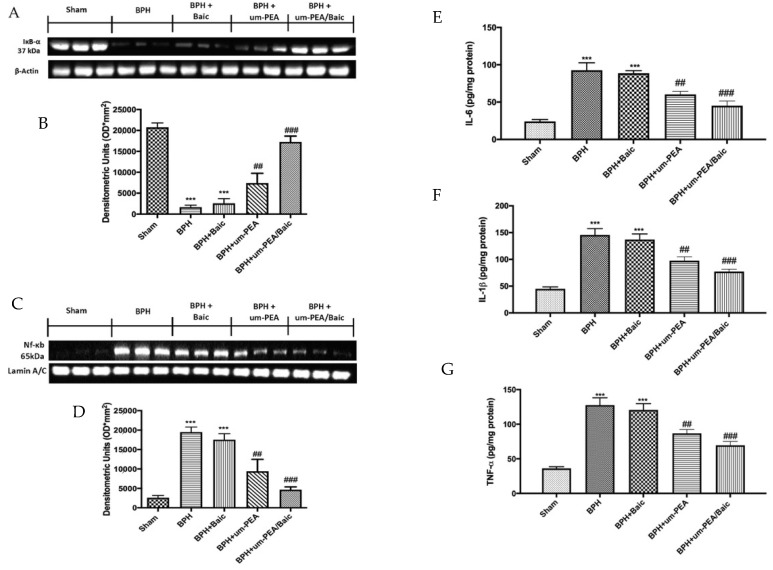
Effect of um-PEA/Baic on inflammation pathway. Western blots and densitometric analysis of IκB-α (**A**,**B**) and NF-κB (**C**,**D**). IL-6 (**E**), IL-1β (**F**) and TNF-α (**G**) levels. A demonstrative blot of lysates with a densitometric analysis for all animals is shown. Values shown are means ± SEM of six animals in each group. *** *p* < 0.001 vs. sham; ## *p* < 0.01 vs. BPH group; ### *p* < 0.001 vs. BPH group.

To investigate how um-PEA/Baic could attenuate the inflammatory process, we investigated the NF-κB pathway by Western blot analysis. Prostate homogenates showed a marked decrease in IκB-α expression in rats after BPH induction compared to the control group that showed a basal expression of IκB-α. At the same time, NF-κB levels were increased significantly in samples from BPH-treated rats. Similar results were obtained in tissues from rats treated with Baic alone. Um-PEA/Baic treatment, more than um-PEA administration alone, reduced IKB-α degradation (Figure 5A) and consequently nuclear translocation of NF-κB (Figure 5C) induced by BPH damage.

In order to determine whether um-PEA/Baic may modulate the secretion of pro-inflammatory cytokines, we also analyzed levels of IL-6, IL-1β and TNF-α (Figure 5E–G, respectively) by ELISA kits. A substantial increase in IL-6, IL-1β and TNF-α was found in the BPH group, while the association of um-PEA/Baic at a dose of 10 mg/kg reduced, in a significant manner, cytokines levels, more than the administration of um-PEA alone. On the contrary, Baic at the dose of 1 mg/kg did not produce any reduction in cytokine production.

### 3.6. Effect of um-PEA/Baic on Oxidative Stress

**Figure 6:**
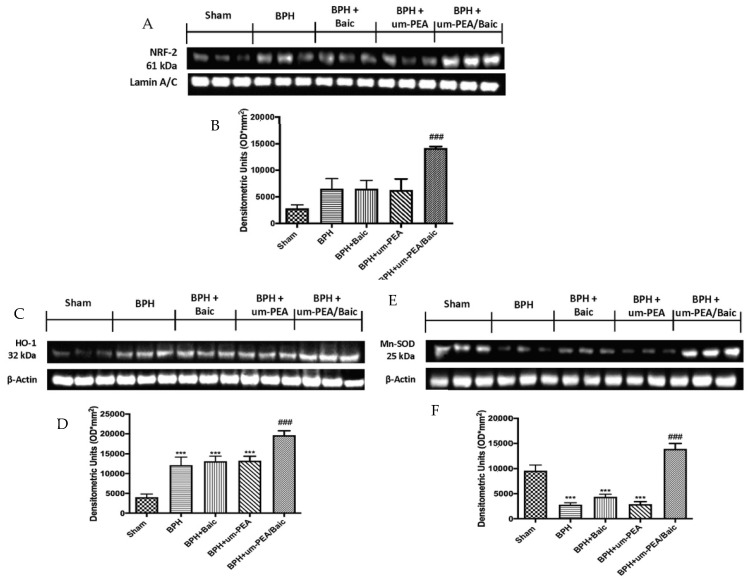
Effect of um-PEA/Baic on oxidative stress. Western blots and densitometric analysis of Nrf-2 (**A**,**B**), HO-1 (**C**,**D**) and Mn-SOD (**E**,**F**). A demonstrative blot of lysates with a densitometric analysis for all animals is shown. Values shown are means ± SEM of six animals in each group. *** *p* < 0.001 vs. sham; ### *p* < 0.001 vs. BPH group.

To understand the molecular mechanisms underlying the antioxidant activity of um-PEA/Baic, we decided to investigate the Nrf2 pathway, which plays a key role in orchestrating cellular antioxidant defenses and in maintaining cellular redox homeostasis. Western blot analysis showed a non-significant increase in the BPH group; similar results were detected in animals after um-PEA and Baic treatment. A considerable increase in Nrf2 expression was induced by um-PEA/Baic administration (Figure 6A,B).

Additionally, HO-1 (Figure 6C,D) and Mn-SOD (Figure 6E,F) are two Nrf2-regulated genes that play a critical role in maintaining antioxidant/oxidant homeostasis [39]. In line with this, a significant increase in levels of both markers was observed in the um-PEA/Baic group, compared to other experimental groups.

## 4. Discussion

Benign prostatic hyperplasia is a non-cancerous enlargement of the prostate that involves hypertrophy of prostatic stromal and epithelial cells. The detailed mechanism of BPH is still unknown, but androgen/androgen receptor (AR) signaling plays a key role in the development of BPH [40]. Testosterone, which is the main circulating androgen, is produced in the testes and moves to the prostate. Here, it is converted to DHT by 5α-red 2, whose isoform 2 is abundantly expressed by prostate tissue, whereas isoform 1 is less specific and more broadly expressed in human tissues [41]. Both testosterone and DHT bind to and activate the AR, but DHT shows two- to five-fold higher affinity in prostate cells and elevates AR signaling 10-fold compared to testosterone [8].

The AR acts as an intracellular nuclear receptor functioning as a ligand-inducible transcription factor [42]. The AR is assumed to be situated in the cytoplasm when it is not coupled with a ligand and thus inactive. After its interaction with androgens, the cytoplasmic AR is activated by a conformational change [43]. This complex molecular unit becomes active and dissociates from the inhibitory heat shock protein 90. The DHT-AR complex is subsequently transported to the nucleus, where it binds to target genes (androgen response elements). It forms a multi-protein complex with coregulators that interacts directly with transcriptional factors and the constitutional transcription machinery to regulate gene transcription [44,45]. Through this mechanism, DHT stimulates the different cell populations of the prostate to produce specific proteins, such as PSA, and a variety of growth-stimulating or growth-inhibiting factors that regulate cell proliferation and function [46]. In fact, it is well known in the literature that the overexpression of PSA causes increased proliferation of prostatic epithelial cells, as indicated by a higher expression of TGF-β [9]. Under physiological conditions, as the stem cells are stimulated by androgens to undergo proliferation and differentiation, the terminally differentiated cells with accumulated damage are removed by apoptosis and a steady-state balance is maintained between cell proliferation and apoptosis. Certain pathological assaults may trigger androgen- and/or growth factor-mediated hyperstimulation of prostate cells, disrupting the normal balance between proliferation and apoptosis [47,48]. As a result, a subset of epithelial cells may evade the normal checkpoint control of cell cycle progression and proliferate aberrantly. Thus, the modulation of AR expression or its signaling may control the development of prostatic hyperplasia or prostate cancer. By blocking AR–androgen binding, anti-androgen drugs—such as bicalutamide and flutamide—cause a decreased prostate volume in BPH patients, despite elevated serum testosterone [49]. However, their utilization for BPH treatment is limited due to a range of sexual side effects, such as erectile dysfunction and ejaculatory changes [50]. Due to the side effects of existing medications, there is a need for alternative natural medicine.

Concerning that, we designed the present study to explore the effects of a new co-ultramicronized compound that combines the anti-inflammatory properties of um-PEA and the antioxidant properties of Baic in an experimental model of testosterone propionate-induced BPH. In line with the above, we clearly showed that oral administrations of um-PEA/Baic at the dose of 10 mg/kg significantly reduced testosterone and DHT levels both in serum and in prostate homogenates. At the same time, um-PEA/Baic was able to reduce the up-regulation of 5α-red 2, AR and PSA expressions, induced by BPH. These data suggest that um-PEA/Baic plays a possible protective role in BPH through modulation of testicular androgens and the AR signaling pathway. Additionally, testosterone injection induced a disruption of the equilibrium between proliferation and apoptosis in prostate tissues. This was proved by the increase in prostate weight and TGF-β expression; on the other hand, prostate tissue collected from BPH rats showed a significant increase in Bcl-2 levels without changes in the expression of Bax compared to sham animals. The co-ultramicronization between um-PEA and Baic in a fixed ratio of 10:1 markedly suppressed the overexpression of TGF-β and reduced levels of anti-apoptotic Bcl-2 while increasing the expression of pro-apoptotic Bax. Changes in the apoptotic pathway (Bcl2 and Bax expressions) were results of DHT reduction in the prostatic tissue due to um-PEA/Baic treatment.

Moreover, prostatic inflammation represents an important factor for BPH development [4,51]. In the present study, histological evaluation revealed intense hyperplasia and pathological alterations of prostate tissues after BPH induction, which were significantly decreased following um-PEA/Baic (10 mg/kg) treatment. Um-PEA/Baic also reduced the number of intact or degranulated mast cells, as shown by toluidine blue staining. This protective effect may be partly due to the down-regulation of the NF-κB pathway, an important mediator of inflammation. In a normal condition, it is bound by the inhibitor protein IkB-α, which sequesters it into the cytoplasm. After the application of external stimuli, IkB-α is degraded, releasing NF-κB from the complex and allowing migration into the nucleus, where it activates translation and transcription of pro-inflammatory mediators [52]. This clarifies the noticed increase in the prostatic content of IL-6, IL-1β and TNF-α after testosterone injection. Conversely, um-PEA/Baic at the dose of 10 mg/kg decreased those inflammatory mediators as well as reducing NF-κB translocation and inhibiting IκBα degradation induced by BHP.

Additionally, it is known that oxidative stress can aggravate the inflammatory responses via activation of NF-κB [53,54]. According to recent studies, Nrf2 regulates the expression of key protective enzymes through the antioxidant response element (ARE) in prostate cancer, and a number of its target genes are down-regulated in human prostate cancer and BPH [55]. Our results show that um-PEA/Baic treatment led to a considerable improvement in Nrf2, as well as increased HO-1 and MnSOD expression levels, suggesting the involvement of the Nrf2 antioxidant pathway in the mechanism of protection of PEA/Baic compared to the action of um-PEA, which showed only anti-inflammatory effects. This up-regulation of Nrf2 by um-PEA/Baic co-administration is due to the synergy that is created between the anti-inflammatory action of um-PEA, which, through the NF-κB pathway, acts indirectly on Nrf2, and the antioxidant properties of Baic.

## 5. Conclusions

In conclusion, our results demonstrate that the new formulation of um-PEA co-ultramicronized with Baic in a fixed ratio of 10:1 modulates the levels of testicular androgens and the AR signaling pathway. Furthermore, the co-administration of um-PEA/Baic creates a synergistic effect by reducing inflammatory processes and oxidative stress in a BPH model, supporting the hypothesis that um-PEA/Baic could be further explored as a valid candidate for the treatment of BPH.

## Data Availability

The datasets used in the current study are available from the corresponding author on reasonable request.
